# Comparative survival analysis of platinum‐based adjuvant chemotherapy for early‐stage squamous cell carcinoma and adenocarcinoma of the lung

**DOI:** 10.1002/cam4.4570

**Published:** 2022-03-10

**Authors:** Shih‐Hsin Hsiao, Wan‐Ting Chen, Chi‐Li Chung, Yu‐Ting Chou, Sey‐En Lin, Shiao‐Ya Hong, Jer‐Hwa Chang, Tzu‐Hao Chang, Li‐Nien Chien

**Affiliations:** ^1^ Division of Pulmonary Medicine, Department of Internal Medicine, School of Medicine, College of Medicine Taipei Medical University Taipei Taiwan; ^2^ Division of Pulmonary Medicine, Department of Internal Medicine Taipei Medical University Hospital Taipei Taiwan; ^3^ Health and Clinical Data Research Center, Office of Data Taipei Medical University Taipei Taiwan; ^4^ Institute of Biotechnology National Tsing Hua University Hsinchu Taiwan; ^5^ Department of Anatomic Pathology New Taipei Municipal Tucheng Hospital, Built and Operated by Chang Gung Memorial Foundation Tucheng New Taipei City Taiwan; ^6^ Medical Research Center, Cardinal Tien Hospital New Taipei City Taiwan; ^7^ Divsion of Pulmonary Medicine Department of Internal Medicine Taipei Municipal Wang Fang Hospital Taipei Taiwan; ^8^ Graduate Institute of Biomedical Informatics, College of Medical Science and Technology Taipei Medical University Taipei Taiwan; ^9^ Clinical Big Data Research Center Taipei Medical University Taipei Taiwan; ^10^ School of Health Care Administration, College of Management Taipei Medical University Taipei Taiwan; ^11^ Health Data Analytics and Statistics Center, Office of Data Science Taipei Medical University Taipei Taiwan

**Keywords:** 5‐year survival rate, early‐stage non–small‐cell lung cancer, overall survival, platinum‐based adjuvant chemotherapy, treatment failure‐free survival

## Abstract

**Background and Purpose:**

Although cytotoxic platinum‐based adjuvant chemotherapy (pACT) has been recommended for patients with completely resected early‐stage (ES) non–small‐cell lung cancer (ES‐NSCLC), therapeutic regimens for NSCLC have evolved in the past two decades. The study was aimed to examine the effectiveness of postoperative pACT for resected ES‐NSCLC patients with squamous cell carcinoma (SCC) or adenocarcinoma (ADC) according to real‐world data.

**Methods and Patients:**

Inverse probability treatment weighting (IPTW) was used to adjust baseline characteristics between the group receiving pACT and those not receiving any treatment (observation, OBS) within 3 months after curative surgery. Cox regression models were used to compare overall survival (OS) and treatment failure‐free survival (TFS) between the groups.

**Results:**

Of 31,208 patients with ES‐NSCLC, 4700 undergoing complete tumor resection were eligible, with a mean follow‐up period of 4.5 years. The pACT (*n* = 2347) and OBS (*n* = 2353) groups were well‐balanced after IPTW. The median OS differed between the pACT and OBS groups (77.2 vs. 75.5 months, adjusted hazard ratio [aHR] = 0.87, 95% confidence interval [CI] = 0.79–0.95, *p* = 0.003), and the 5‐year survival rates were 58.2% and 55.3%, respectively (*p* < 0.001). In the SCC group, pACT was superior to OBS in OS (75.0 vs. 57.4 months, aHR = 0.74, 95% CI = 0.62–0.88, *p* = 0.001) and TFS (32.7 vs. 21.8 months, aHR = 0.74, 95% CI = 0.63–0.86, *p* < 0.001). Both OS and TFS did not differ between two groups in those with ADC.

**Conclusion:**

Real‐world data indicated that pACT confers a survival benefit for resected ES‐NSCLC patients with SCC but not ADC, which needs to be verified by a large sample of randomized controlled studies.

## Graphical abstract

Cytotoxic platinum‐based adjuvant chemotherapy (pACT) is recommended for patients with completely resected early‐stage (ES) non–small‐cell lung cancer (ES‐NSCLC). However, this real‐world data indicated that pACT confers a survival benefit for patients with ES‐NSCLC with SCC but not ADC.

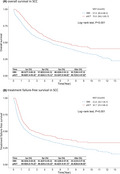

## 1 INTRODUCTION

Lung cancer is the most common cause of cancer‐related deaths worldwide and is conventionally classified into small‐cell lung cancer (SCLC) and non–small‐cell lung cancer (NSCLC).^1^ Although 25% of NSCLC cases diagnosed at the early stage (ES; stage I–IIIA) were potentially curable through curative surgery, approximately 30%–40% of these patients developed tumor recurrence in other sites in the body.^2^, ^3^ The 5‐year survival rate (5YSR) of ES‐NSCLC was around 50%.^3^, ^4^, ^5^, ^6^ Cytotoxic platinum‐based adjuvant chemotherapy (pACT) has often been suggested to patients with completely resected ES‐NSCLC after curative surgery since the mid‐2000s.^7^, ^8^, ^9^ However, a pooled analysis of the five largest randomized controlled trials (RCTs) revealed that the survival benefit of pACT was modest (hazard ratio [HR] = 0.89) with an absolute increase of 5.4% in the 5YSR.^10^ Many adverse effects (AEs) including fatigue, anorexia, alopecia, vomiting, neutropenia, infection, and death (0.9%) caused by cytotoxic pACT have been reported.^10^ Moreover, the International Adjuvant Lung Cancer Trial found that cytotoxic pACT was associated with a late increase in cytotoxic pACT‐related mortality.^6^

Therapeutic regimens for recurrent or metastatic NSCLC have remarkably evolved in the past two decades. Studies published in the 2000s have reported that the efficacy of new cytotoxic antineoplastic agents against advanced NSCLC depends on the subtypes of NSCLC such as adenocarcinoma (ADC) and squamous cell carcinoma (SCC), which are two main subtypes.(^11^, ^12^, ^13^, ^14^, ^15^) Pemetrexed‐based therapy when administered as both first‐line and second‐line treatments resulted in higher overall survival (OS) in patients with advanced nonsquamous NSCLC (most of whom had ADC) than in their counterparts (HR: 0.78 and 0.84, respectively) but not in patients with squamous NSCLC (HR: 1.56 and 1.23, respectively).^11^ Moreover, targeted therapy (e.g., epidermal growth factor receptor [EGFR] tyrosine kinase inhibitors) has been used to treat recurrent and advanced NSCLC since 2003^16^, ^17^ and is the most effective in the ADC subtype, particularly in tumors with EGFR mutations.^18^ Antiangiogenic agents (e.g., bevacizumab) combined with platinum‐based doublet chemotherapy showed a survival benefit (HR for death = 0.79; *p* = 0.003) in recurrent and metastatic NSCLC^19^; however, its clinical application is only approved in the nonsquamous subtype.^20^

Based on the aforementioned findings, whether cytotoxic platinum‐based pACT provides an increased survival advantage in both patients with resected lung ES‐SCC and ES‐ADC in the daily clinical setting remains unclear. RCTs are often conducted under restrictive conditions (e.g., good performance status and few comorbidities) and intensive monitoring by specialized research personnel and completed within a relatively short timeframe.^7^, ^8^, ^9^ The use of real‐world evidence (RWE) has become a complementary source to RCT data for obtaining more robust evidence‐based treatment effectiveness in clinical practice.^21^ Therefore, the present study examined the survival benefit of cytotoxic platinum‐based pACT in patients with completely resected ES‐NSCLC in real‐world settings and hypothesized that its survival advantage differs between SCC and ADC subtypes.

## 2 MATERIALS AND METHODS

### 2.1 Ethics statement

This study was approved by the Joint Institutional Review Board of Taipei Medical University (approval no. N202102077).

### 2.2 Study design and database

In this retrospective cohort study, three administrative databases from Taiwan were used to perform the analysis. The Taiwan Cancer Registry (TCR) is a population‐based reporting system that tracks patients with a cancer diagnosis. All hospitals are mandated to submit cancer data to the TCR under the legislation of the Cancer Control Act, 2003, including detailed information regarding cancer diagnosis and first‐course treatment.^22^ The population‐based National Health Insurance (NHI) Research Database (NHIRD) contains the claims data of patients enrolled in Taiwan's NHI program, including health care services and drug prescriptions.^23^ Since 1995, all the residents of Taiwan are required by law to enroll in the NHI, resulting in a coverage rate of over 99%. The NHI program reimburses most medical expenses for patients with cancer, indicating that whether these patients received medical services for their disease largely depends on their willingness rather than financial considerations. The National Death Registry (NDR) is also a population‐based registry that contains cause‐of‐death data. The three datasets can be linked by unique encrypted identifiers under the regulation of the Health and Welfare Data Science Center of the Ministry of Health and Welfare in Taiwan.

### 2.3 Study cohort

**FIGURE 1:**
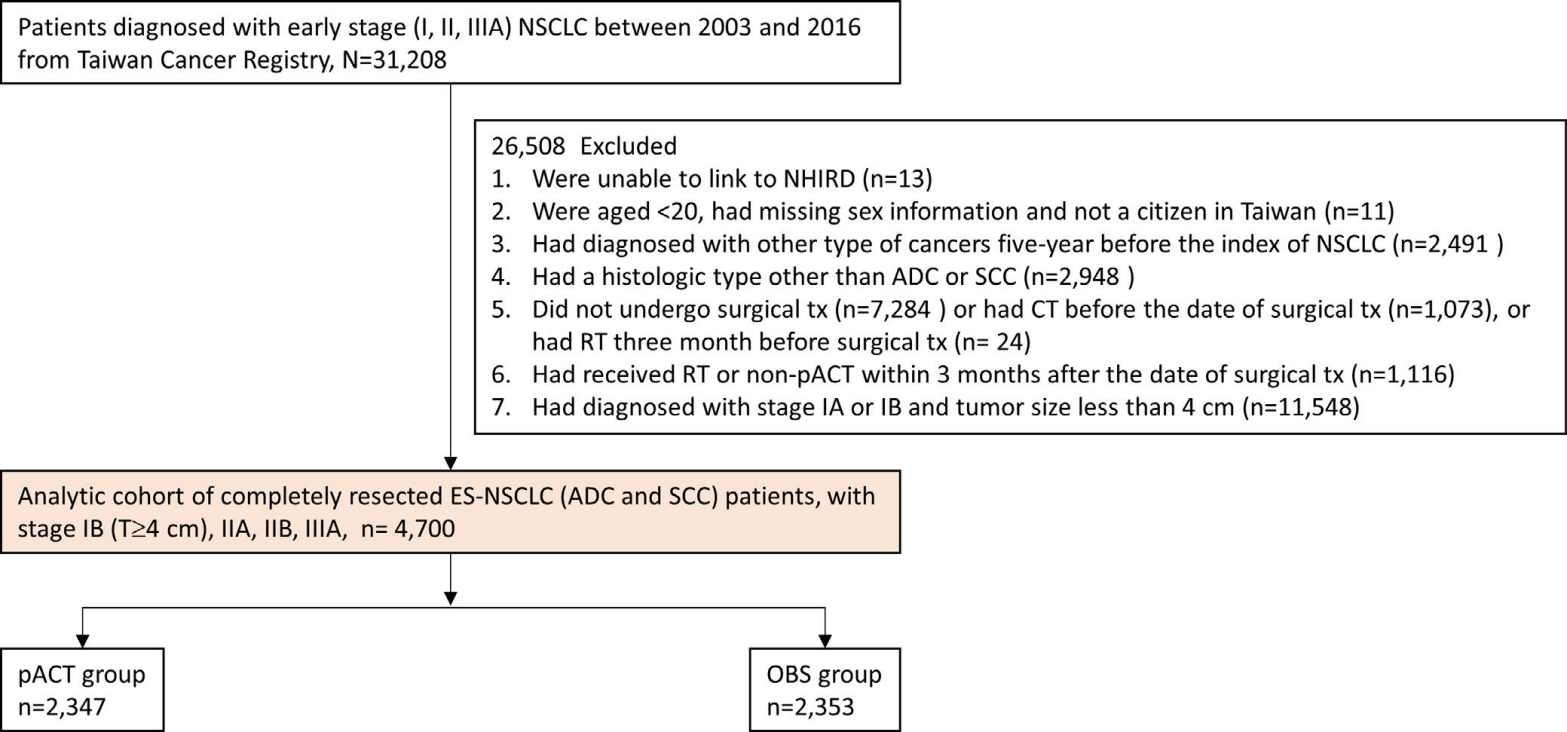
Patient selection process. Abbreviations: ADC, adenocarcinoma; CT, chemotherapy; ES‐NSCLC, early‐stage non–small‐cell lung cancer; NHIRD, National Health Insurance Research Database; OBS, observation; pACT, platinum‐based adjuvant chemotherapy; RT, Radiotherapy; SCC, squamous cell carcinoma; tx, treatment

From the TCR, we identified patients who were histologically or cytologically diagnosed as having ES (I, II, and IIIA, defined by the American Joint Committee on Cancer, 6th edition from 2003 to 2009 and 7th edition from 2010 to 2016) NSCLC between January 2003 and December 2016. We excluded patients who (1) were unable to be linked to the NHIRD; (2) were aged <20 years or had missing sex information and not a citizen in Taiwan; (3) had other cancers within 5 years before the diagnosis of lung cancer; (4) had a subtype other than SCC or ADC; (5) did not receive surgical treatment, had received chemotherapy (CT) or radiotherapy (RT) before surgery; (6) received RT within 3 months after surgery; and (7) were diagnosed as having stage IA or IB (tumor size <4 cm) (Figure 1). Among eligible patients, those who received pACT after their curative surgery were included in the pACT group, whereas those who did not receive any antineoplastic treatment (e.g., drugs and RT) within 3 months after their curative surgery were included in the observation group (hereafter referred to as the OBS group). Figure 1 presents the detailed patient selection process.

### 2.4 Outcome definition

The outcome of interest was OS, which was defined as the period from the index date of surgical resection to the date of death. The death record was obtained from the NDR. Treatment failure‐free survival (TFS) was defined as the interval from the date of the surgery to the date of anti‐lung cancer therapy (including CT, target therapy, RT, and surgery) or the date of death which came first. All patients were followed up from the index date until the occurrence of outcomes or December 31, 2018.

### 2.5 Study variables

Information regarding sex, age, histological type, and cancer staging was obtained from the TCR. For patients who were diagnosed as having cancer after 2011, we could obtain the Eastern Cooperative Oncology Group Performance Score (ECOGPS). Data regarding the surgical procedure were obtained from the NHIRD. In addition, we used the Deyo–Charlson comorbidity index (DCCI) to adjust the severity of comorbidities derived from medical claims records in the NHIRD. Detailed codes for disease diagnosis, surgical procedures, and medication use are listed in Table S1.

### 2.6 Statistical analysis

Several factors might be associated with the effectiveness of pACT, resulting in significant differences in baseline characteristics between the two groups. Therefore, we used inverse probability treatment weighting (IPTW) to adjust the imbalance. IPTW is a method based on propensity scoring used to balance baseline variables without sample loss. IPTW is regarded as an appropriate method for estimating treatment effects on time‐to‐event outcomes.^24^, ^25^ Each patient was weighted by stabilized IPTW after the propensity score was generated. This method has been widely adopted in many observational studies. The standardized mean difference (SMD) was calculated, and SMD > 0.1 indicates the presence of a non‐negligible difference between the pACT and OBS groups.

A Kaplan–Meier survival curve was plotted to compare the overall OS and TFS of the two groups, and the log‐rank test was performed. Multivariable Cox proportional hazard regression analysis was performed to estimate the effect of pACT on the outcomes of interest. For analyses, patients were divided into ADC and SCC groups. None of the models violated the assumption of the proportional hazard. In addition, we conducted several subgroup analyses including for sex, age, cancer staging, and diagnostic year as well as ECOGPS. All analyses were performed using SAS/STAT 9.2 (SAS Institute Inc, Cary, NC.). Statistical significance was set at *p* < 0.05.

## 3 RESULTS

### 3.1 Baseline characteristics

Of 31,208 patients with ES‐NSCLC, 4700 (15.1%) were eligible for inclusion in this study (Figure 1). Among eligible patients, 2475 (52.7%) were alive on December 31, 2018. Table 1 lists the baseline characteristics of the pACT (*n* = 2347) and OBS (*n* = 2353) groups before IPTW. In particular, patients in the pACT group were younger, predominantly being diagnosed with cancer between 2014 and 2016 and had ADC as the histological type, had the advanced‐stage disease (IIB–IIIA), and had lower DCCI scores. The two groups were well‐balanced after IPTW. For example, 71.1% and 72.6% of patients in the ACT and OBS groups, respectively, had the ADC subtype (SMD = 0.032). Notably, the mean (±standard deviation [SD]) follow‐up periods did not differ between the two groups (4.5 ± 3.2 vs. 4.4 ± 3.0 years). The mean number of pACT cycles in the pACT group was 3.24 (SD = 1.07).

**TABLE 1:** Baseline characteristics of patients with completely resected ES‐NSCLC receiving platinum‐based adjuvant chemotherapy (pACT) versus no treatment (OBS) after surgery

Variables	Before IPTW			After IPTW		
	OBS (*n* = 2353)	pACT (*n* = 2347)	SMD	OBS (*n* = 2353)	pACT (*n* = 2347)	SMD
	(%)	(%)		(%)	(%)	
Male	60.4	53.1	0.147	56.6	56.5	0.003
Age (y), mean ± SD	67.5 ± 10.9	60.9 ± 9.8	0.638	64.8 ± 11.2	64.2 ± 10.3	0.051
20–54	12.6	25.1	0.322	18.8	18.9	0.004
55–64	23.0	35.7	0.283	29.5	29.5	<0.001
65+	64.4	39.2	0.521	51.7	51.6	0.003
Year of diagnosis						
2003–2006	10.0	6.5	0.126	8.2	8.3	0.002
2007–2010	29.7	22.2	0.172	25.1	24.7	0.009
2011–2014	39.7	43.1	0.070	41.8	42.3	0.009
2015–2016	20.7	28.2	0.176	24.9	24.8	0.003
Histological type						
ADC	68.6	79.3	0.244	74.1	74.1	<0.001
SCC	31.4	20.7	0.244	25.9	25.9	<0.001
Cancer staging						
IB + IIA	54.8	32.6	0.460	43.7	43.4	0.006
IIB + IIIA	45.2	67.4	0.460	56.3	56.6	0.006
Type of surgery						
Pneumonectomy or bilobectomy	7.0	5.8	0.050	6.3	6.4	0.003
Lobectomy	77.8	80.4	0.063	78.6	78.4	0.005
Wedge resection	15.2	13.9	0.038	15.1	15.2	0.004
DCCI, mean ± SD	1.4 ± 1.4	1.0 ± 1.2	0.293	1.2 ± 1.4	1.2 ± 1.3	0.031
0	29.3	41.2	0.250	35.5	35.5	<0.001
1	34.1	34.9	0.016	34.3	34.6	0.006
2+	36.6	24.0	0.277	30.2	29.9	0.006
ECOGPS^a^						
Missing	49.9	37.5	0.251	43.0	42.5	0.011
0	29.5	40.3	0.228	35.8	35.9	0.002
1+	20.6	22.2	0.038	21.2	21.6	0.010
Follow‐up period (y), mean ± SD	4.5 ± 3.2	4.4 ± 2.7	0.023	4.4 ± 3.0	4.5 ± 2.8	0.029

*Note*: Values are presented as the percentage or mean ± SD. SMD > 0.1 indicates the presence of a non‐negligible difference between the two groups.

Abbreviations: ADC, adenocarcinoma; DCCI, Deyo–Charlson comorbidity index; ECOGPS, Eastern Cooperative Oncology Group Performance Score; ES‐NSCLC, early‐stage non–small‐cell lung cancer; IPTW, inverse probability treatment weighting; OBS, observation; pACT, platinum‐based adjuvant chemotherapy; SCC, squamous cell carcinoma; SD, standard deviation; and SMD, standardized mean difference.

^a^ ECOGPSs were obtained after the year 2011.

### 3.2 Kaplan–Meier curve for OS and TFS

**FIGURE 2:**
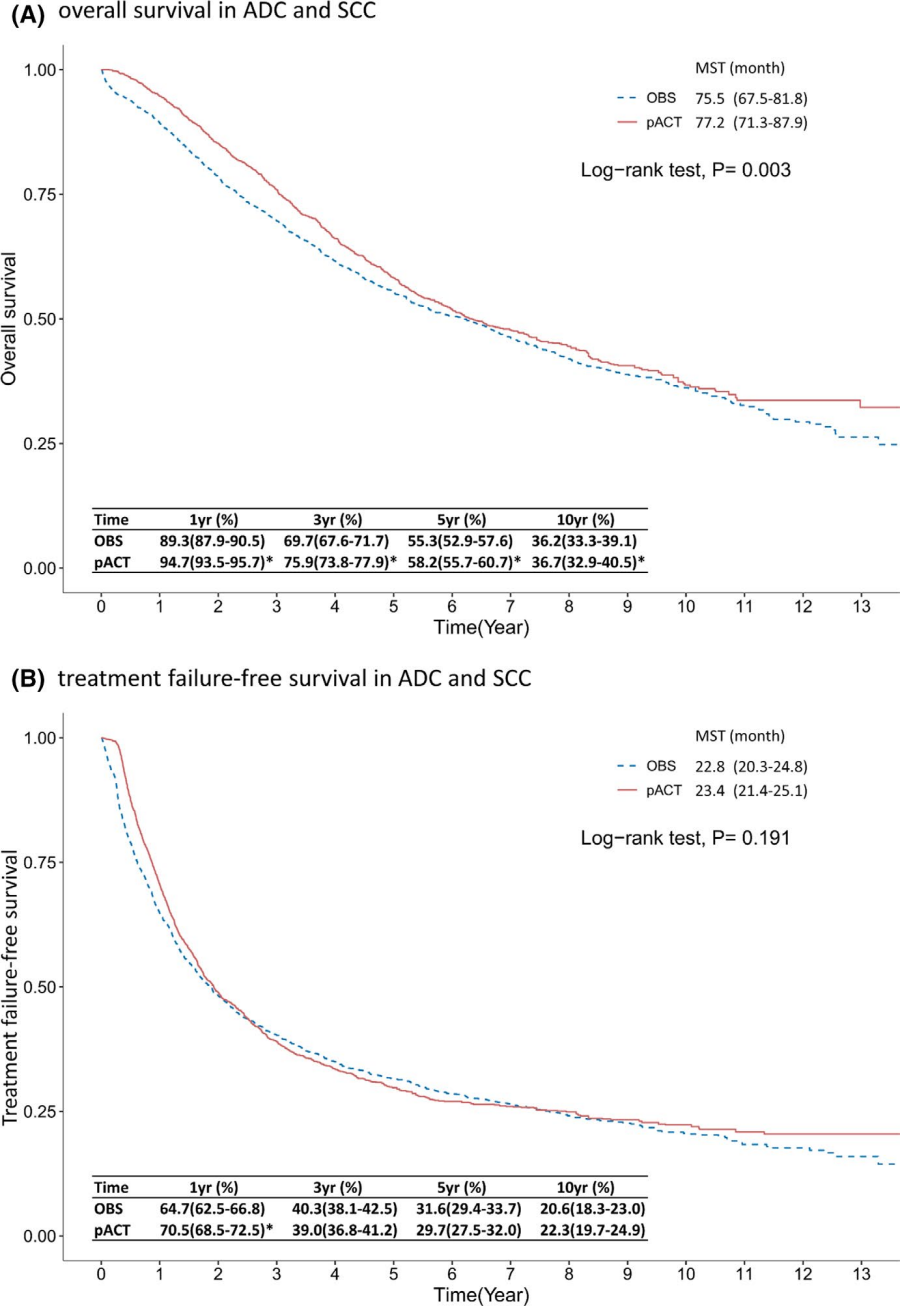
Kaplan–Meier survival curve of (A) overall survival and (B) treatment failure‐free survival of patients with completely resected ES‐NSCLC receiving pACT versus OBS. *indicates a difference between the two groups in the beneficial effect of pACT on outcomes. Abbreviations: ES‐NSCLC, early‐stage non–small‐cell lung cancer; MST, median survival time; OBS, observation; pACT, platinum‐based adjuvant chemotherapy; yr, year

As indicated in Figure 2A, the median survival time (MST) was higher in the pACT group (77.2 vs. 75.5 months, *p* = 0.003 for log‐rank test), and the pACT group also had a higher 5YSR than did the OBS group (58.2% vs. 55.3%, *p* < 0.001). However, we did not observe significantly higher TFS in the pACT group (23.4 vs. 22.8 months of MST, *p* = 0.191 in Figure 2B), and the pACT group exhibited only a significantly higher 1‐year TFS rate (70.5% vs. 64.7%) but not 5‐year TFS rate (29.7% vs. 31.6%) than did the OBS group.

**FIGURE 3:**
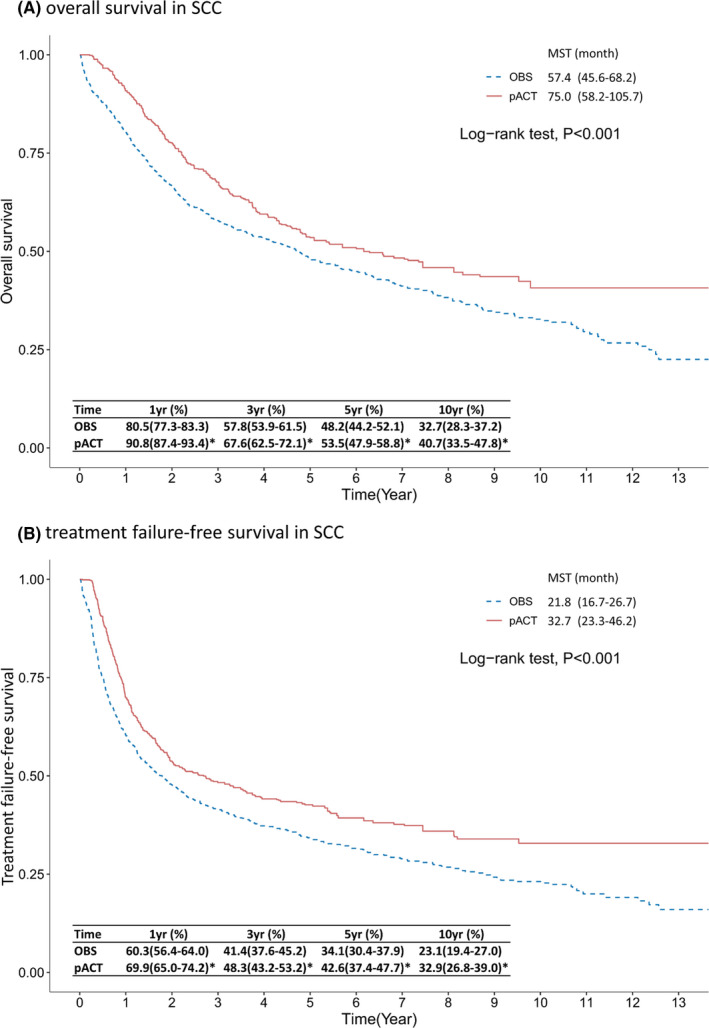
Kaplan–Meier survival curve of (A) overall survival and (B) treatment failure‐free survival of patients with completely resected ES‐NSCLC SCC receiving pACT versus OBS. *indicates a difference between the two groups in the beneficial effect of pACT on outcomes. Abbreviations: ES‐NSCLC, early‐stage non–small‐cell lung cancer; MST, median survival time; OBS, observation; pACT, platinum‐based adjuvant chemotherapy; SCC, squamous cell carcinoma; yr, year

**FIGURE 4:**
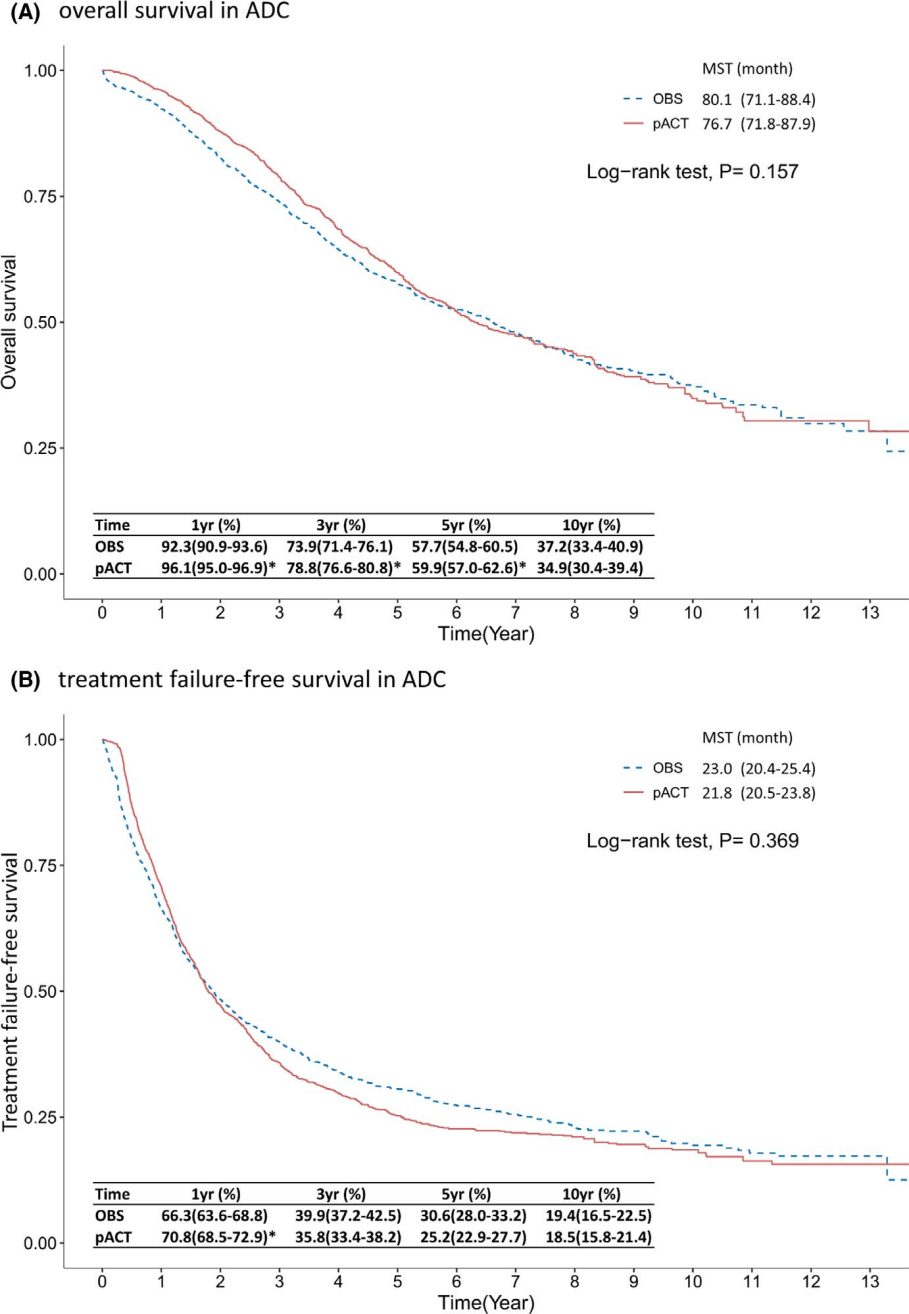
Kaplan–Meier survival curve of (A) overall survival and (B) treatment failure‐free survival of patients with completely resected ES‐NSCLC ADC receiving pACT versus OBS. * indicates a difference between the two groups in the beneficial effect of pACT on outcomes. Abbreviations: ADC, adenocarcinoma; ES‐NSCLC, early‐stage non–small‐cell lung cancer; MST, median survival time; OBS, observation; pACT, platinum‐based adjuvant chemotherapy; yr, year

Regarding the SCC subtype of resected ES‐NSCLC, the results revealed that OS was significantly different between the pACT and OBS groups (75.0 vs. 57.4 months of MST, *p* < 0.001 in Figure 3A), and the 5YSR of the pACT group was significantly higher than that of the OBS group (53.5% vs. 48.2%, *p* < 0.001). TFS also differed between the pACT and OBS groups (32.7 vs. 21.8 months, *p* < 0.001), and the pACT group had higher TFS rates than did the OBS group at various follow‐ups (e.g., 42.6% vs. 34.1% at 5 years in Figure 3B). Regarding the ADC subtype of resected ES‐NSCLC, the 5YSR of the pACT group was higher than that of the OBS group (59.9% vs. 57.7%, *p* < 0.001); however, this difference was relatively small, and the MST of OS was similar between the two groups (76.7 vs. 80.1 months, *p* = 0.157 in Figure 4A). The median TFS did not differ between the pACT and OBS groups (21.8 vs. 23.0 months, *p* = 0.369 in Figure 4B), and the survival rates were not different except for the 1‐year TFS rate (70.8% vs. 66.3%). Similar results were observed when the ADC subtype group was further classified into stage IB (≥ 4 cm)‐IIA and IIB‐IIIA subsets (Figures S1 and S2).

### 3.3 Cox regression analysis

After we adjusted for covariates listed in Table 1, namely age, sex, tumor stage, year of cancer diagnosis, and DCCI scores, the results of multivariable Cox regression analysis indicated that pACT was significantly associated with a lower risk of death in patients with overall resected ES‐NSCLC (HR = 0.87, 95% CI = 0.79–0.95, *p* = 0.003) and patients with the SCC subtype (HR = 0.74, 95% CI = 0.62–0.88, *p* = 0.001; Table 2). A similar result was obtained when evaluating the outcome of recurrence or death in the SCC group (HR = 0.74, 95% CI = 0.63–0.86, *p* < 0.001). However, the survival benefit of pACT was not significant in the ADC group (HR for death = 0.92, 95% CI = 0.83–1.03, *p* = 0.149; HR for recurrence or death = 1.04, 95% CI = 0.95–1.13, *p* = 0.392).

**TABLE 2:** Multivariable cox regression analysis of all‐cause death and disease recurrence or death of patients with completely resected ES‐NSCLC receiving pACT or no treatment (OBS) after IPTW

Study group	Outcomes	Treatment	Adjusted^a^ HR	(95% CI)	*p*
Overall	All‐cause death	OBS	1.00	(Ref.)	
		pACT	0.87	(0.79–0.95)	0.003
	Disease recurrence or death	OBS	1.00	(Ref.)	
		pACT	0.95	(0.88–1.03)	0.191
SCC	Death	OBS	1.00	(Ref.)	
		pACT	0.74	(0.62–0.88)	0.001
	Disease recurrence or death	OBS	1.00	(Ref.)	
		pACT	0.74	(0.63–0.86)	<0.001
ADC	Death	OBS	1.00	(Ref.)	
		pACT	0.92	(0.83–1.03)	0.149
	Disease recurrence or death	OBS	1.00	(Ref.)	
		pACT	1.04	(0.95–1.13)	0.392

Abbreviations: ADC, adenocarcinoma; ES‐NSCLC, early‐stage non–small‐cell lung cancer; HR, hazard ratio; IPTW, inverse probability treatment weighting; OBS, observation; pACT, platinum‐based adjuvant chemotherapy; SCC, squamous cell carcinoma.

^a^ Adjusted HR was estimated by controlling for baseline characteristics listed in Table 1.

### 3.4 Subgroup analysis

**FIGURE 5:**
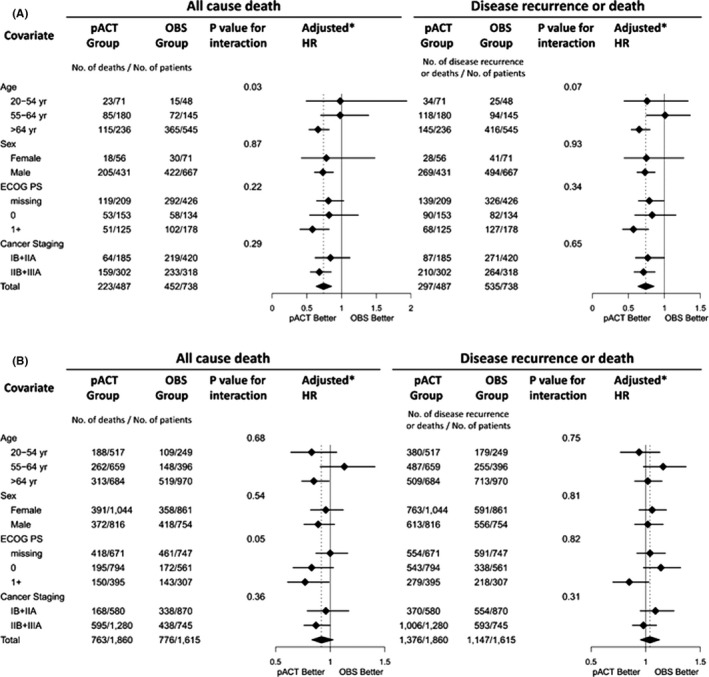
Subgroup analysis of the effectiveness of pACT on the risk of death or recurrence and death of patients with ES‐NSCLC with SCC (A) and ADC (B) subtypes. Abbreviations: ADC, adenocarcinoma; DCCI, Deyo–Charlson comorbidity index; ECOGPS, Eastern cooperative oncology group performance score; ES‐NSCLC, early‐stage non–small‐cell lung cancer; OBS, observation; pACT, platinum‐based adjuvant chemotherapy; SCC, squamous cell carcinoma

Among patients with SCC, the subsets of patients aged over 64 years, male patients, those with an ECOGPS of 1+, and those diagnosed as having stage IIB–IIIA disease benefited from the administration of cytotoxic pACT in the outcome of all‐cause death (Figure 5A, Left). Similarly, the benefit of pACT in terms of recurrence or death was observed (Figure 5A, Right). The subgroup analysis of patients with ADC revealed that the pACT only benefited a few patient subsets such as those aged over 64 years, those with an ECOGPS of 1+, and those with stage IIB–III disease (Figure 5B, Left). None of the patient subsets benefited from the pACT in terms of recurrence or death (Figure 5B, Right).

### 3.5 Toxicity of pACT

Since neutropenia was the most frequently noted pACT‐related toxicity, we used the frequency of granulocyte‐colony stimulating factor (G‐CSF) administration, which is routinely administered because of grade 3–4 severe neutropenia (defined as an absolute neutrophil count less than 1000/μL) during patients' pACT course, as a surrogate of severe pACT‐related AEs. The analysis showed that 128 (5.5%) of 2347 patients who received pACT required G‐CSF treatment (288 events) during the pACT course.

## 4 DISCUSSION

This study based on nationwide data obtained from real‐world settings confirmed the survival advantage of the administration of cytotoxic pACT in patients with overall completely resected ES‐NSCLC. Because some patients were followed up for over 10 years, we observed that the survival advantage of pACT declined over time and was not robust at 5 and 10 years, with survival rates difference of 2.9% and 0.7%, respectively (Figure 2A). Furthermore, our analysis revealed that pACT provided a significant benefit for patients with the SCC subtype in terms of OS, TFS, and 5YSR; however, the advantage of pACT in terms of the 5YRS in patients with the ADC subtype was small, and both the median OS and TFS were not significantly different between the pACT and OBS groups.

A direct comparison of the results of real‐world data (RWD) with those of previous RCTs in which patients were enrolled from 1994 to 2001 according to strict criteria and protocols for patient care often precisely followed by a well‐organized expert team is difficult.^7^, ^8^, ^9^ In the current study, we used almost the same major criteria of RCTs to select patients from real‐world settings. However, the baseline characteristics of our cohort were heterogeneous and not well‐balanced between the pACT and OBS groups before the IPTW adjustment (Table 1). Therefore, we used the IPTW method to adjust the baseline difference between the two groups and confirmed the OS benefit of pACT in patients with completely resected ES‐NSCLC; this result is consistent with the finding of previous RCTs that revealed that the OS benefit of patients receiving pACT was 4.1%–15.0% in the 5YSR, and the results of the pooled analyses of five large RCTs indicated an absolute increase of 5.4% in the 5YSR.^10^

To date, no RCT has suggested that histology alone can serve as a criterion for identifying patients with completely resected ES‐NSCLC who would benefit from postoperative pACT.^10^ There is also no nationwide dataset‐based study that examined the benefit of pACT among resected ES‐NSCLC patients. Recently, two small institute‐based studies have shown that pACT was associated with improved disease‐free survival (DFS) and OS of resected ES‐NSCLC in the real‐world setting,^26^, ^27^ however, they did not further investigate whether the survival benefit of pACT differed between ES‐SCC and ES‐ADC groups. Compared to RCTs, this current study was able to obtain a relatively bigger sample size with more heterogeneous groups; however, the interpretation should be cautious because the smaller effect size was also more likely to be observed.

The findings of the current cohort study revealed that patients with the SCC subtype benefited from pACT in terms of OS, and the survival advantage persisted up to 10‐year follow‐up. By contrast, the survival advantage of pACT for patients with the ADC subtype was not dominant in terms of OS, declined over time, and was relatively small. The finding suggested that, in general, patients with ADC did not live longer despite receiving a mean of 3.24 cycles of pACT and experienced pACT‐related AEs including severe neutropenia (5.5%). Compared with patients with resected ES‐SCC, those with ADC had more opportunities for benefiting from the progress of therapeutic regimens for recurrent and metastatic NSCLC,^14^, ^15^, ^16^, ^17^, ^18^, ^19^, ^20^ irrespective of whether they received postoperative pACT. Collectively, these RWD and RWE suggest that patients with the SCC subtype may be more appropriate candidates for postoperative cytotoxic pACT because they tend to exhibit greater benefit in terms of improved OS than do patients with the ADC subtype in daily clinical practice.

The most common theory for the need for postoperative systemic adjuvant therapy for completely resected NSCLC is that some occult micrometastases or dormant cancer cells elsewhere in the body that cannot be detected before surgery can proliferate and form tumors in distant organs at some point after surgery, leading to patients’ death. Thus, the eradication or suppression of these cancer cells can prevent tumor recurrence. Our data showed pACT confers survival benefits for patients with resected ES‐SCC but not ES‐ADC. The full mechanism to explain this finding is unclear; however, three potential reasons may partially explain it. First, the in vitro study showed the chemosensitivity to SCC was better than it to ADC,^28^ although no data have demonstrated whether chemosensitivity to dormant cells from primary ES‐SCC or ES‐ADC is different. Second, lung ADC tends to metastasize to the brain than SCC does, and the concentration of chemotherapy‐platinum in the central nervous system fluid is very low (around 2 to 10% of that in the plasma).^29^, ^30^ Thus, the effect of pACT on DFS (TFS in this study) or OS, could be, theoretically, different among resected ES‐SCC and ES‐ADC groups. Third, the profound advance of therapeutic regimens for post‐surgery recurrent tumors focused mostly on ADC during the study period (2003–2016), therefore, the effect of pACT on OS in resected ES‐ADC groups could be diminished by the novel antineoplasm drugs.

Genotyping was not routinely suggested for patients with resected ES‐NSCLC during 2003–2016, thus the current study was unable to answer whether pACT provided different survival advantages for patients with distinct actionable genetic alterations (e.g., EGFR mutations, ALK, or ROS1 fusions). To our best knowledge, whether the survival effect of pACT on patients with resected ES‐NSCLC is influenced by the underlying driver genotypes is not determined by large RCT trials. More recently, gefitinib, a first‐generation EGFR tyrosine kinase inhibitor (TKI), has been shown its advantage on DFS but not OS of resected ES‐NSCLC patients (most ADC≥90%) with EGFR mutations when compared with pACT.^31^, ^32^ Osimertinib, a third‐generation EGFR TKI, could prolong the DFS of this population with common sensitive EGFR mutations,^33^ however, its survival advantage on OS remains unknown. Taken together, additional studies are needed to accurately dissect patients with resected NSCLC, especially ADC, harboring distinct genotypes and biomarkers and identify who would benefit from postoperative adjuvant therapy, including pACT, target therapy, immunotherapy, or combined in the future.^34^

### 4.1 Strengths and limitations

To the best of our knowledge, this is the first study to examine the effectiveness of pACT in terms of OS and DFS among patients of pure Asian ethnicity with ES‐NSCLC by using population‐based RWD. In this study, we included data from a cancer registry and medical claims database that contained detailed information regarding patients’ characteristics, related medical information, and treatment prescriptions, thus reducing the potential bias due to observed confounders. Moreover, because the characteristics of the study cohort were heterogeneous, they reflected the reality in daily practice, enabling us to examine the effectiveness of pACT in various subgroups.

This study has several limitations. First, in observational studies, confounding by indication is an often intractable threat to validity because patients with poor prognosis are more likely to be treated aggressively. We observed that patients who received pACT generally had an advanced disease stage but were younger (Table 1); thus, some confounding by indication was present in this study. We adopted IPTW, multivariable regression models, and subgroup analysis to improve the validity of our findings. Because no adjustment methods could fully resolve the problem of confounding by indication, the results should be implemented and interpreted with caution. Second, the definition of TFS was not fully equivalent to the definition of DFS, therefore, the true effect of pACT on the prevention of tumor recurrence could not be accurately assessed in this study, and TFS might have been overestimated in those patients who did not receive treatment for recurrence in this dataset‐based study. Third, we could not fully adjust the analysis for patients who received medication that was not reimbursed because information regarding out‐of‐pocket health expenditure is not accessible. Moreover, we analyzed medical expenditure and found that the medical cost was significantly higher in patients with ADC who received pACT than in those who were observed (Table S2). We did not analyze the effect of pACT on patients’ quality of life because relevant information was not routinely collected in the databases we used in the current study. Finally, because data were derived from people of Asian ethnicity, the results may not be generalizable to other populations.

## 5 CONCLUSION

The considerable progress in cancer histological or genomic typing and treatment for recurrent and metastatic tumors is shaping the care of both patients with metastatic and ES‐NSCLC. Our RWE revealed that patients with SCC may be more appropriate candidates for postoperative pACT than patients with ADC in terms of improved OS which needs to be verified by a large sample of RCTs. The results of this study are a first step toward the development of treatment that can be selected more appropriately based on histological type and biomarkers. Additional studies classifying patients with ES‐ADC who harbor different driver oncogenes into subsets are warranted to identify those who would benefit from postoperative cytotoxic pACT.

## CONFLICT OF INTEREST

The authors have no potential conflicts of interest to report.

## AUTHOR CONTRIBUTIONS

Shih‐Hsin Hsiao involved in conceptualization, methodology, and writing—original draft preparation of the manuscript. Wan‐Ting Chen carried out software preparation of the manuscript. Chi‐Li Chung, Yu‐Ting Chou, Sey‐En Lin, Shiao‐Ya Hong, and Jer‐Hwa Chang involved in writing—reviewing and editing of the manuscript. Tzu‐Hao Chang and Li‐Nien Chien involved in supervision, conceptualization, methodology, and writing—original draft preparation of the manuscript.

## Supporting information


Figure S1

Figure S2

Table S1

Table S2

DataS1

## Data Availability

In regard to data availability, our study used healthcare administrative data that provided by Health and Welfare Data Science Center (HWDC), Ministry of Health and Welfare in Taiwan. The HWDC is a third‐party organization. Researchers can submit application to HWDC in order to have access to several health‐related databases. Due to legal restrictions imposed by the government of Taiwan in relation to the Personal Information Protection Act, data cannot be made publicly available. Requests for data can be sent as a formal proposal to the HWDC with an IRB approval for research purpose only. The contact information of Taipei Medical University Joint IRB is tmujirb@gmail.com. All data were fully anonymized before we access them. In addition, these data can only be access and analyzed in an independent operating area in the HWDC. And only statistical results can be brought out from the operating area. Therefore, original data cannot be shared publicly due to legal restrictions.
