# Supplementary figures and images for: Comparative survival analysis of platinum‐based adjuvant chemotherapy for early‐stage squamous cell carcinoma and adenocarcinoma of the lung
Source: Cancer Med. 2022 Mar 10;11(10):2067–78. doi: 10.1002/cam4.4570 (PMC9119352; doi:10.1002/cam4.4570)

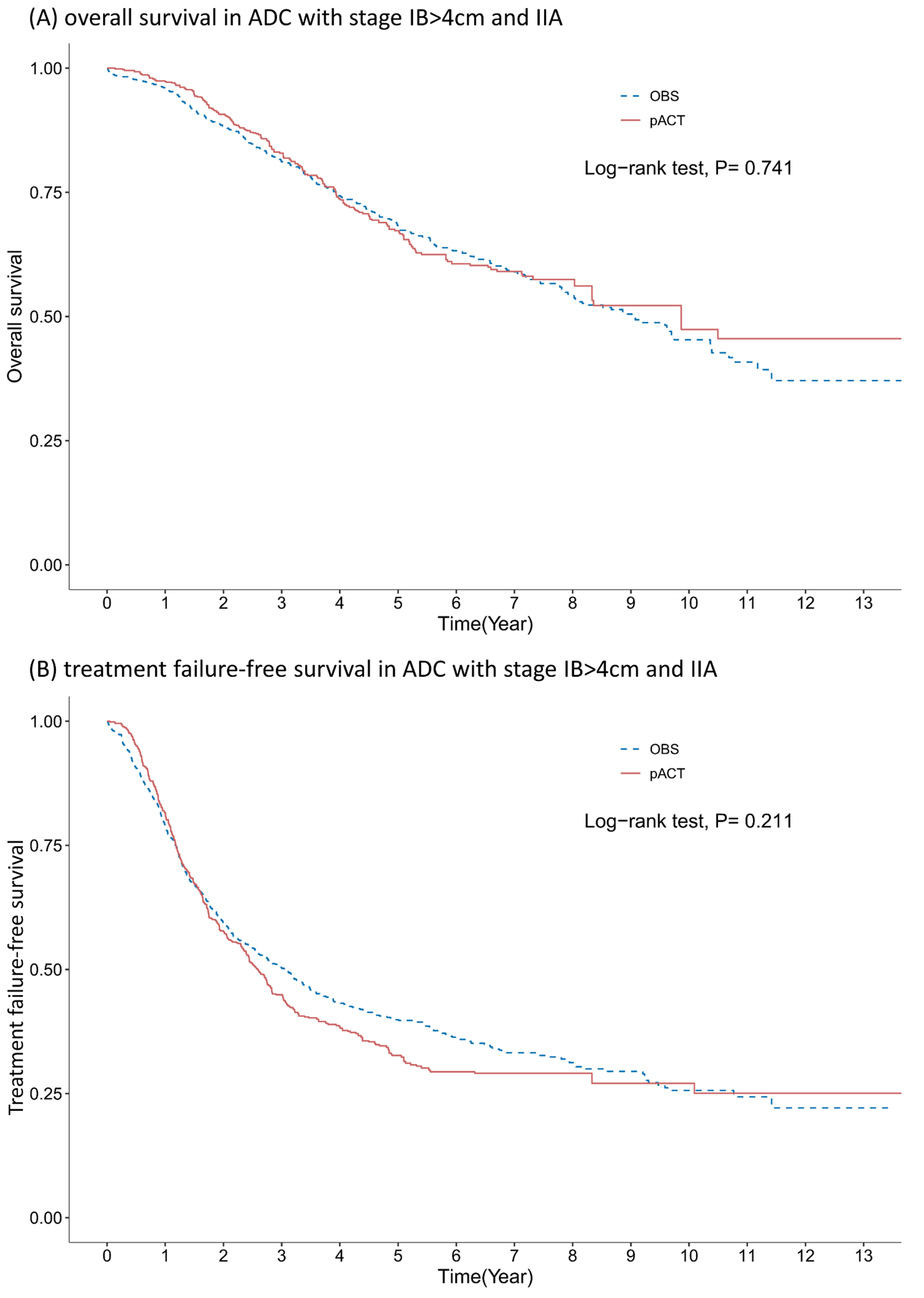

Supplement: Supplementary file 1 — Figure S1 [file CAM4-11-2067-s001.tif]

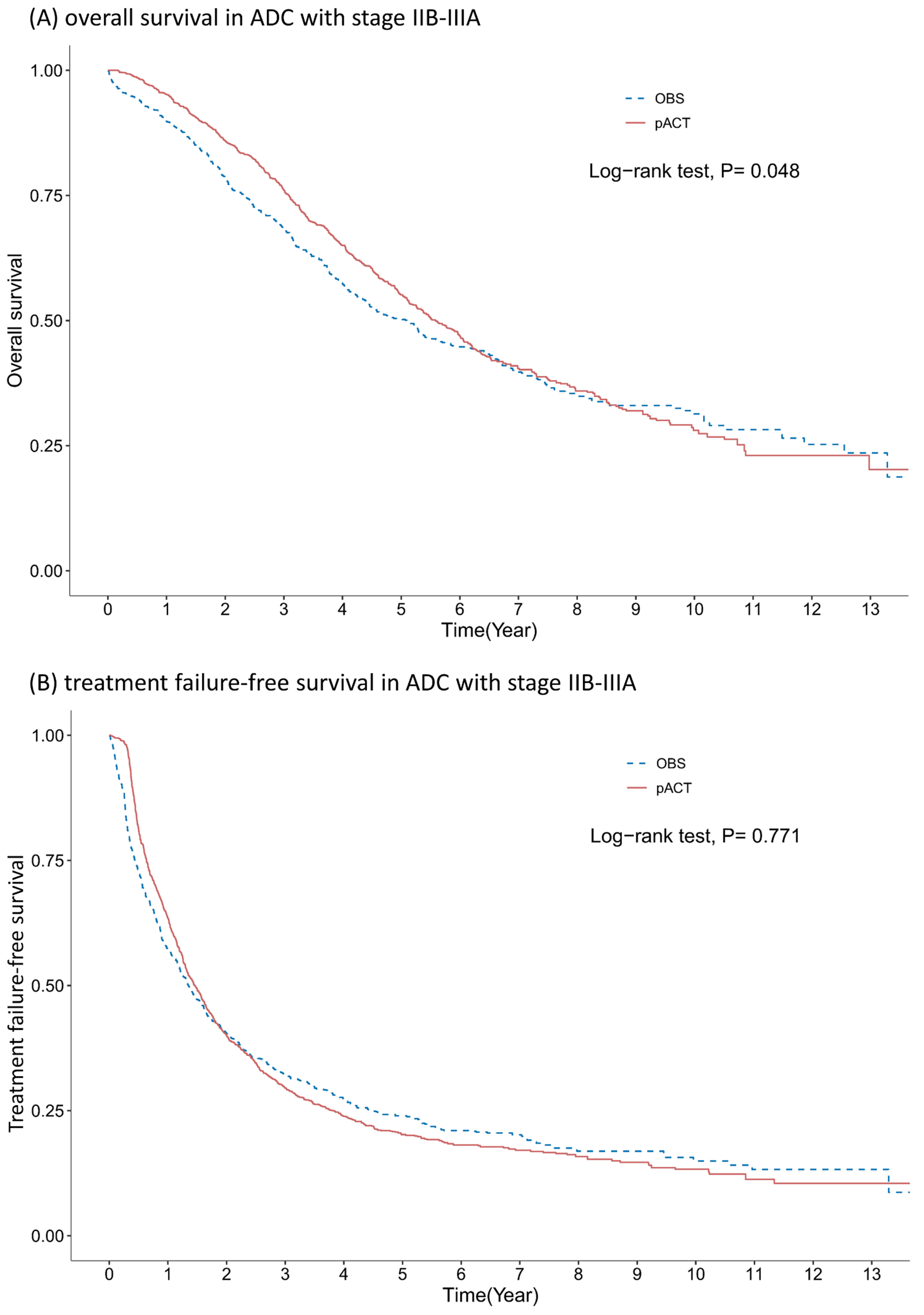

Supplement: Supplementary file 2 — Figure S2 [file CAM4-11-2067-s004.tif]
