# Supplementary material for: Comparative survival analysis of platinum‐based adjuvant chemotherapy for early‐stage squamous cell carcinoma and adenocarcinoma of the lung
Source: Cancer Med. 2022 Mar 10;11(10):2067–78. doi: 10.1002/cam4.4570 (PMC9119352; doi:10.1002/cam4.4570)
Supplement: Supplementary file 3 — Table S1 [file CAM4-11-2067-s005.docx]

| Appendix Table 1. ICD-O-3 morphology codes for histology, ATC codes for chemotherapy, and NHI procedure codes for surgery and radiotherapy | |
| --- | --- |
| Histological type |  |
| ADC | 8050 , 8140 , 8141 , 8143 , 8201 , 8230 , 8250 , 8251 , 8252 , 8253 , 8254 , 8255 , 8256 , 8257 , 8260 , 8265 , 8310 , 8320 , 8323 , 8480 , 8481 , 8490 , 8550 , 8551 , 8572 |
| SCC | 8052, 8070, 8071, 8072, 8073, 8074, 8076, 8082,8083 |
| Surgical type |  |
| Pneumonectomy | 67024B, 67049B, 67053B |
| Bilobectomy | 67042B |
| Lobectomy | 67023B, 67029B, 67050B, 67054B |
| Wedge resection | 67051B |
| Treatment |  |
| Radiotherapy | 36011B, 36012B |
| pACT | L01XA02, L01XA01 |
| Chemotherapy | L01CD02 , L01CB01 , L01BC05 , L01CD01 , L01CD03 , L01BA04 , L01XX17 , L01CA01 , L01CA04 , L01AA01 , L01DB01 , L01XX19 , L01BC52, |
| Targeted therapy | L01XE03 , L01XE02 , L01XE13 , L01XE16 , L01XC07 |

Abbreviations: ADC = adenocarcinoma; ATC = Anatomical Therapeutic Chemical; ICD-O-3 = *International Classification of Disease for Oncology, third edition*; NHI = National Health Insurance; pACT = platinum-based adjuvant chemotherapy; SCC = squamous cell carcinoma
