# Supplementary material for: Comparative survival analysis of platinum‐based adjuvant chemotherapy for early‐stage squamous cell carcinoma and adenocarcinoma of the lung
Source: Cancer Med. 2022 Mar 10;11(10):2067–78. doi: 10.1002/cam4.4570 (PMC9119352; doi:10.1002/cam4.4570)
Supplement: Supplementary file 4 — Table S2 [file CAM4-11-2067-s002.docx]

| Appendix Table 2. Medical expenditure ($, per person per year) of patients with ES-NSCLC who received surgical treatment with pACT and OBS | | | | | | | | | | | | | | | |
| --- | --- | --- | --- | --- | --- | --- | --- | --- | --- | --- | --- | --- | --- | --- | --- |
|  |  | **Before IPTW** | | | | | | | **After IPTW** | | | | | | |
|  |  | **OBS (n = 2353 )** | | | **pACT (n = 2347)** | | | **K–W *P*** | **OBS (n = 2353 )** | | | **pACT (n = 2347)** | | | **K-W *P*** |
| Group | Medical expenditure | Median | Q1 | Q3 | Median | Q1 | Q3 |  | Median | Q1 | Q3 | Median | Q1 | Q3 |  |
| Overall | Overall cost | 10,030 | 4,143 | 19,088 | 12,757 | 5,703 | 19,847 | <0.001 | 10,165 | 4,088 | 18,882 | 12,519 | 5,456 | 19,687 | <0.001 |
|  | Cancer-related cost | 7,199 | 2,241 | 16,199 | 11,047 | 4,224 | 18,222 | <0.001 | 7,512 | 2,294 | 16,587 | 10,747 | 3,966 | 17,894 | <0.001 |
| ADC | Overall cost | 10,065 | 4,288 | 18,109 | 13,276 | 6,051 | 19,813 | <0.001 | 10,217 | 4,151 | 18,208 | 13,241 | 6,051 | 19,701 | <0.001 |
|  | Cancer-related cost | 7,586 | 2,401 | 15,610 | 11,711 | 4,483 | 18,224 | <0.001 | 7,867 | 2,403 | 16,268 | 11,438 | 4,338 | 17,955 | <0.001 |
| SCC | Overall cost | 9,871 | 3,878 | 22,572 | 9,615 | 4,789 | 20,577 | 0.960 | 9,875 | 3,752 | 22,791 | 9,457 | 4,721 | 19,573 | 0.820 |
|  | Cancer-related cost | 6,251 | 1,995 | 18,472 | 7,518 | 3,470 | 18,183 | 0.013 | 6,173 | 1,999 | 18,521 | 7,341 | 3,149 | 17,651 | 0.069 |

Abbreviation: ADC = adenocarcinoma; IPTW = inverse probability treatment weighting; K–W P= Kruskal–Wallis test *P* value; ES-NSCLC = early-stage non–small-cell lung cancer; OBS = observation; pACT = platinum-based adjuvant chemotherapy; SCC = Squamous cell carcinoma

30 NTD = 1 USD
