# Supplementary material for: Comparative survival analysis of platinum‐based adjuvant chemotherapy for early‐stage squamous cell carcinoma and adenocarcinoma of the lung
Source: Cancer Med. 2022 Mar 10;11(10):2067–78. doi: 10.1002/cam4.4570 (PMC9119352; doi:10.1002/cam4.4570)
Supplement: Supplementary file 5 — DataS1 [file CAM4-11-2067-s003.docx]

**Appendix Figure legend**

**Appendices Fig 1: Kaplan–Meier survival curve of (A) overall survival and (B) treatment failure-free survival of patients with NSCLC ADC stage IB > 4 cm and IIA who received surgical treatment with pATC versus OBS**

Abbreviations: ADC = adenocarcinoma; NSCLC = non–small-cell lung cancer; OBS = observation; pACT = platinum-based adjuvant chemotherapy

**Appendices Fig 2: Kaplan–Meier survival curve of (A) overall survival and (B) treatment failure-free survival of patients with NSCLC ADC stage IIB–IIIA receiving surgical treatment with pATC versus OBS**

Abbreviations: ADC = adenocarcinoma; NSCLC = non–small-cell lung cancer; OBS = observation; pACT = platinum-based adjuvant chemotherapy
